# Supplementary material for: CRISPR/Cas12-Based Ultra-Sensitive and Specific Point-of-Care Detection of HBV
Source: Int J Mol Sci. 2021 May 3;22(9):4842. doi: 10.3390/ijms22094842 (PMC8125043; doi:10.3390/ijms22094842)
Supplement: Supplementary file 1 [file ijms-22-04842-s001.zip › ijms-1185815-revised-r1-supplementary/Supplementary materials.pdf]

Supplementary information

Figure S1 ROC curve of Cas12a-DETECTR assay

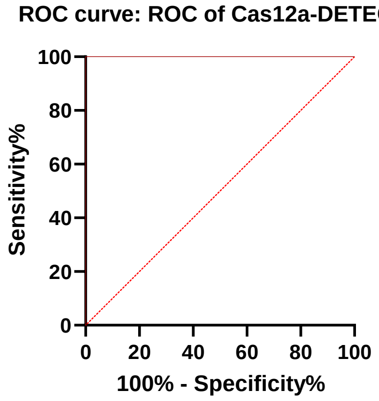

In vitro transcription (IVT) steps of crRNA

I Synthesis of double-stranded crRNA

Table S1 First add to the PCR tube according to the system:

Component	Volume to add (μL)
crRNA template, 100 μM	1
T7 primer, 100 μM	1
RNase-Free Water	8
Total	10

Next, anneal the crRNA template and T7 primers by performing a 10 min denaturation. The reaction was then slowly cooled to 4°C in a PCR thermocycler.

II T7 transcription of crRNA

Table S2 The above products were mixed in the following system:

Component	Volume to add (μL)
Annealing reaction	10
NTP buffer mix	10
T7 RNA polymerase mix	2
RNase-Free Water	17
Total	39

The HiScribe™ T7 Quick High Yield RNA Synthesis Kit (NEB, E2050S) was mixed according to the above system and placed in a 37°C incubator for 10 hours.

III Purification of crRNA

The above T7 transcript product was purified by using Monarch® RNA Cleanup Kit (10 μg) (NEB, T2030L) and following the instructions strictly.

Table S3 LAMP reaction systems

Component	Volume to add (μL)
2 x LAMP Mix	12.5
FIP, 10μM	2
BIP, 10μM	2
LF, 10μM	1
LB, 10μM	1
F3, 10μM	0.5
B3, 10μM	0.5
DNA polymerase	0.5
HBV DNA	5
Total	25

LAMP reaction condition is 65°C, after the reaction is finished, store on ice

Table S4 Reaction system of Cas12a-DETECTR

Component	Volume to add (μL)
LAMP amplification product	5-10
10 x reaction buffer	2.5
crRNA, 30ng/μL	1.25
Cas12a, 1μM	2
Fluorescent reporter, 10μM or biotin reporter, 1μM	1
RNase-Free Water	Up to 25

The fluorescence assay was performed by mixing the above system and then performing a fluorescence collection assay. However, the lateral flow test strip (Milenia HybriDetect 1, TwistDx) need to be incubated for 15 min at 37°C in a warm metal bath, and then 50 μL of HybriDetect assay buffer is pipetted and mixed to reach the required volume of lateral flow test strip. After 2-3 minutes, the results can be visible to the naked eye.

The optimized Cas12a reaction system is to increase the LAMP amplification product to 10ul, in addition to the lateral flow test strips can also extend the incubation time to 30min.

Table S5. Sequences involved in this study

HBV standard plasmid synthesis

Name	Sequence (5'-3')
Target dsDNA HBV polymerase coding region (MN683731)	TGGTTCTTCTGGACTATCAAGGTATGTTGCCCGTTTGTCCTCTAATTCCAG<br>GATCTTCAACCACCAGCACAGGACCCTGCAGAACCTGCACGACTCCTGC<br>TCAAGGAACCTCTATGTATCCCTCCTGTTGCTGTACAAAACCTTCGGACG<br>GAAATTGCACCTGTATTCCCATCCCATCATCCTGGGCTTTCGGAAAATTC<br>CTATGGGAGTGGGCCTCAGCCCGTTTCTCTTGGCTCAG<u>TTTA</u>**CTAGTGC**<br>**CATTTGTTCAGTG**GTTCGTAGGGCTTTCCCCCACTGTTTGGCTTTCAGT<br>TATATGGATGATGTGGTATTGGGGGCCAAGTCTGTACAGCATCTTGAGTC<br>CCTTTTTACCGCTGTTACCAATTTTCTTTTGTCTTTGGGTATACATTTGAA<br>CCCTAATAAAACCAAACGTTGGGGCTATTCCCTTAACTTCATGGGATATAT

crRNAs

crRNA1	UAAUACGACUCACUAUAGGGUAAUUUCUACUAAGUGUAGAUCUAGUGCCAUUUGUUCAGUG
crRNA2	UAAUACGACUCACUAUAGGGUAAUUUCUACUAAGUGUAGAUUUCAGUGGUUCGUAGGGCUU
T7 primer	TAATACGACTCACTATAGGG
Template crRNA1	CACTGAACAAATGGCACTAGATCTACACTTAGTAGAAATTACCCTATAGTGAGTCGTATTA
Template crRNA2	AAGCCCTACGAACCACTGAAATCTACACTTAGTAGAAATTACCCTATAGTGAGTCGTATTA

LAMP primers

Primer 1	Sequence (5'-3')
F3	GCACCTGTATTCCCATCC
B3	CAAGATGCTGTACAGACTTG
FIP	GAGCCAAGAGAAACGGGCTGCATCATCCTGGGCTTTCG
BIP	AGTGGTTCGTAGGGCTTTCCCCCAATACCACATCATCCAT
LF	GGCCCACTCCCATAGGAATTTTC
LB	CCCACTGTTTGGCTTTCAGT
Primer 2	
F3	TTCCCATCCCATCATCCT
B3	GGTAACAGCGGTAAAAAGG
FIP	GCACTAGTAAACTGAGCCAAGAGAAGGGCTTTCGGAAAATTCC
BIP	CTTTCCCCCACTGTTTGGCTCAAGATGCTGTACAGACTTG
LF	GGCTGAGGCCCACTCCCATA
LB	GGATGATGTGGTATTGGGGGC
Primer 3	
F3	GCACCTGTATTCCCATCC
B3	GGTAACAGCGGTAAAAAGG
FIP	ACTGAGCCAAGAGAAACGGG-CATCATCCTGGGCTTTCG
BIP	CTTTCCCCCACTGTTTGGCT-CAAGATGCTGTACAGACTTG
LF	CTGAGGCCCACTCCCATAGG
LB	GATGATGTGGTATTGGGGGC

ssDNA reporter

Fluorescent reporter	5′-/6-FAM/TTTTTT/BHO/-3′
Biotin reporter	5′-/6-FITC/TTTTTTT/Biotin/-3′

Table S6 Information on purchased reagents

Name	Company and item number
LbCas12a protein	GUANGZHOU BIO-LIFESCI, M20301
HiScribe™ T7 Quick High Yield RNA Synthesis Kit	NEB, E2050S
Monarch® RNA Cleanup Kit (10 μg)	NEB, T2030L
RNase-Free Water	CWBIO, CW0612

2X Lamp PCR Master Mix (Universal)	Shanghai Sangon Biotech,
Milenia HybriDetect 1	Milenia biotec, MGHD1
Reporter	Shanghai Sangon Biotech
HBV Nucleic Acid Assay Kit (Prominence HBV DNA)	Sansure Biotech
Ultra Rapid Nucleic Acid Releaser	Ample Future, WLDR8202
