# Supplementary figures and images for: CRISPR/Cas12-Based Ultra-Sensitive and Specific Point-of-Care Detection of HBV
Source: Int J Mol Sci. 2021 May 3;22(9):4842. doi: 10.3390/ijms22094842 (PMC8125043; doi:10.3390/ijms22094842)

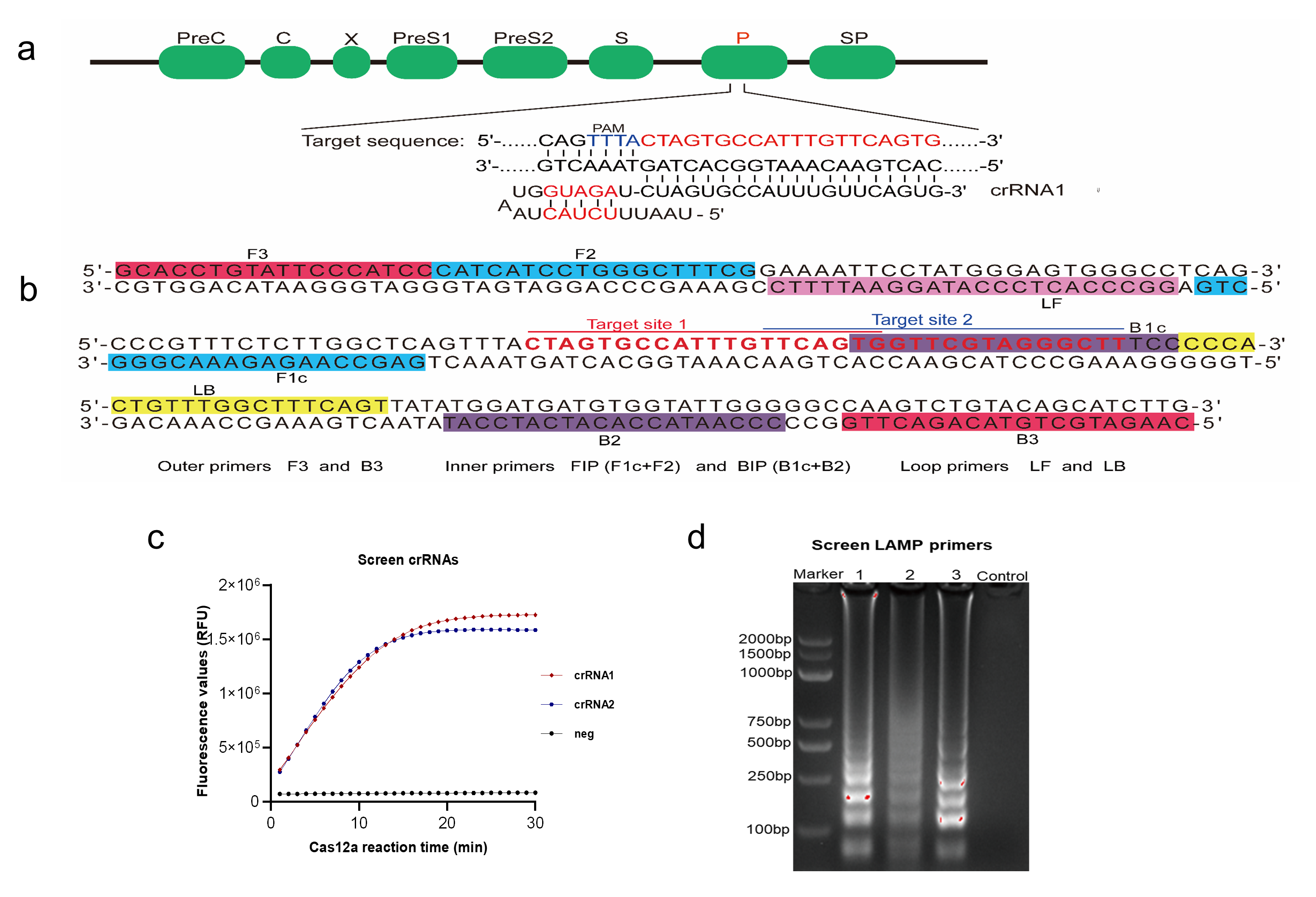

Supplement: Supplementary file 1 [file ijms-22-04842-s001.zip › ijms-1185815-revised-r1-supplementary/Figures/figure 2.tif]

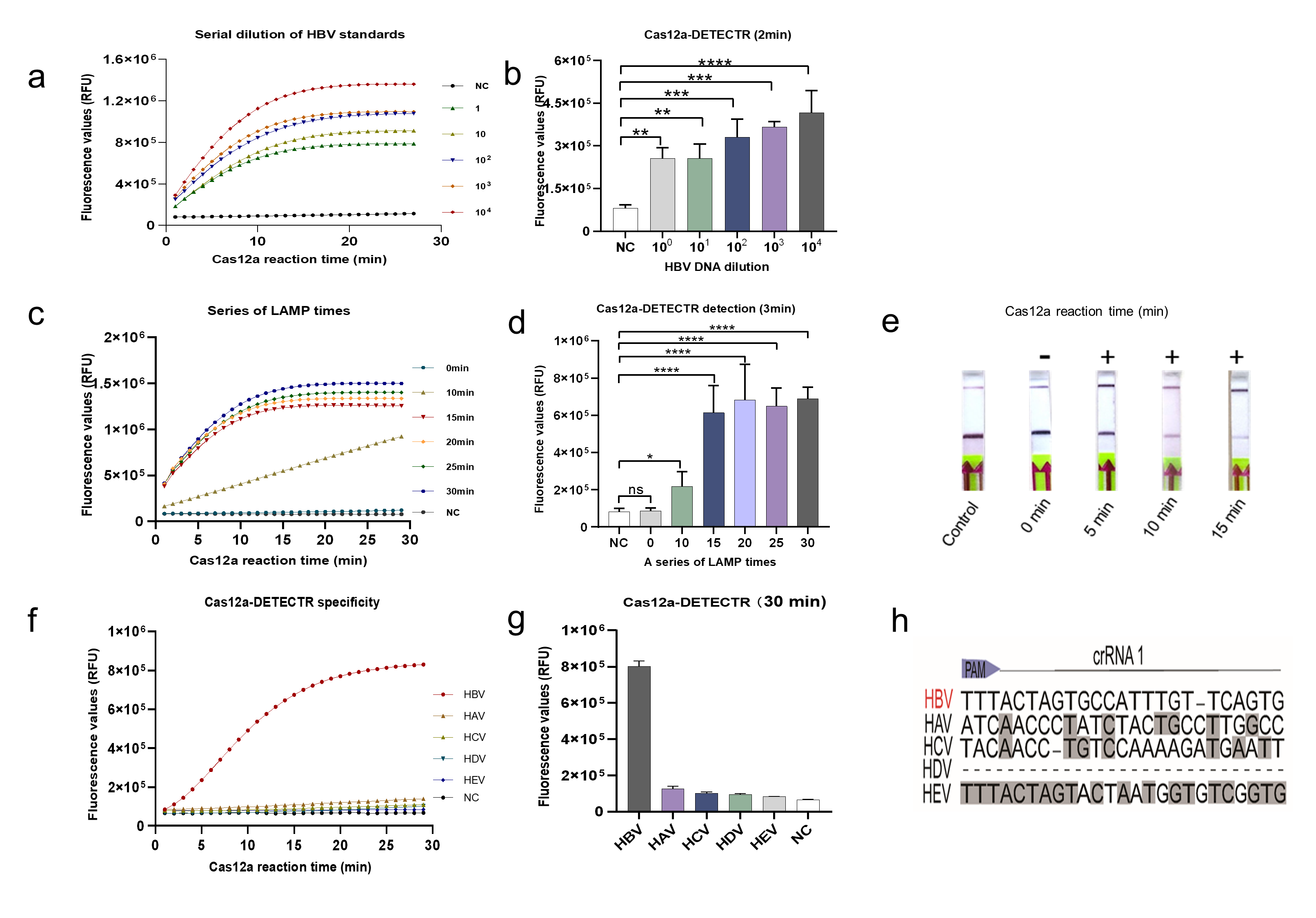

Supplement: Supplementary file 1 [file ijms-22-04842-s001.zip › ijms-1185815-revised-r1-supplementary/Figures/figure 3.tif]

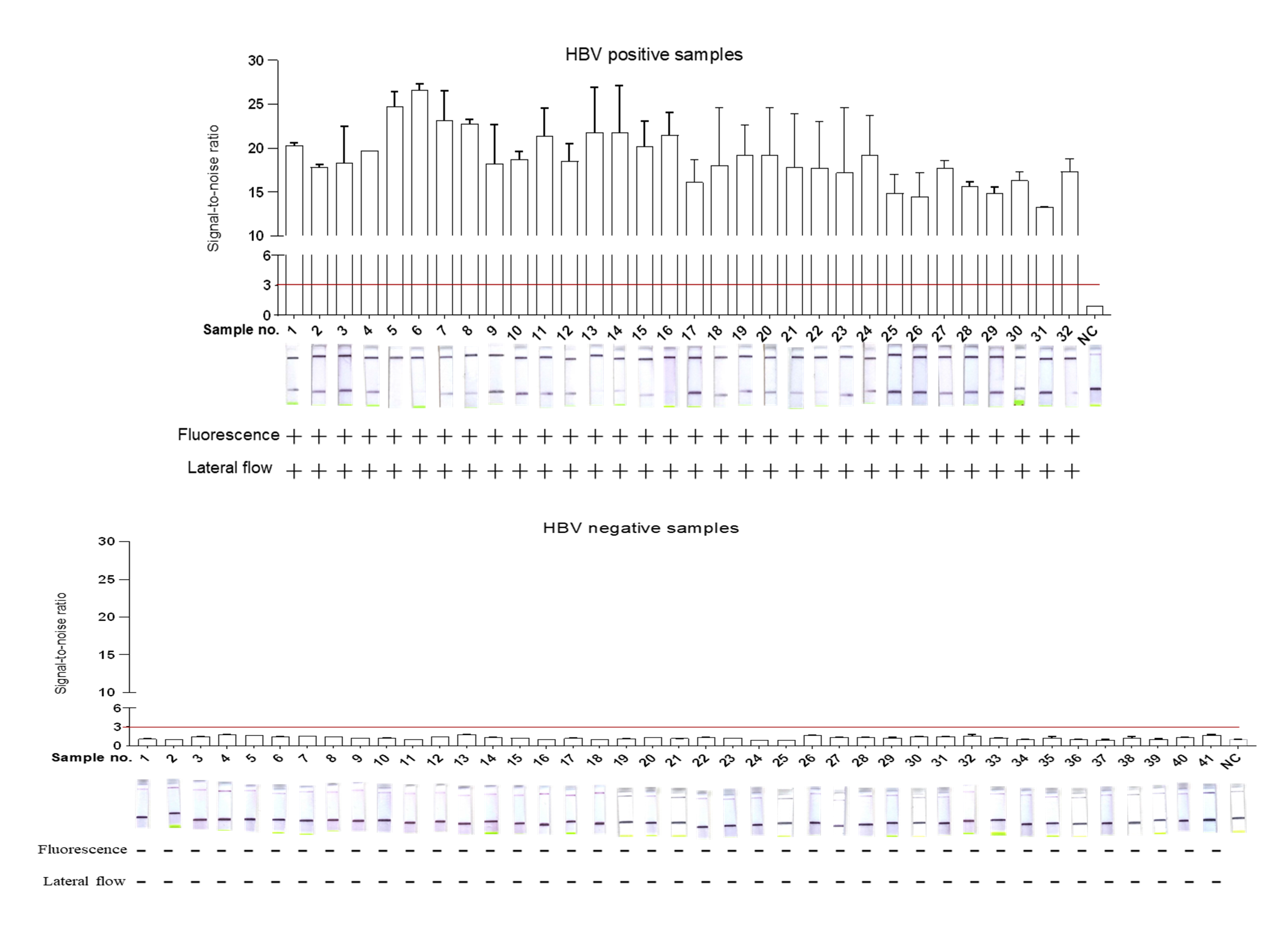

Supplement: Supplementary file 1 [file ijms-22-04842-s001.zip › ijms-1185815-revised-r1-supplementary/Figures/figure 4.tif]
